# Research progress on Mesenchymal Stem Cells (MSCs), Adipose-Derived Mesenchymal Stem Cells (AD-MSCs), Drugs, and Vaccines in Inhibiting COVID-19 Disease

**DOI:** 10.14336/AD.2020.0711

**Published:** 2020-10-01

**Authors:** Pietro Gentile, Aris Sterodimas, Jacopo Pizzicannella, Claudio Calabrese, Simone Garcovich

**Affiliations:** ^1^Department of Surgical Science, University of Rome “Tor Vergata”, Rome, 00133, Italy.; ^2^Academy of International Regenerative Medicine & Surgery Societies, Geneva, Switzerland.; ^3^Department of Plastic and Reconstructive Surgery, Metropolitan General Hospital, Athens, Greece.; ^4^ASL02 Lanciano-Vasto Chieti, Ss. Annunziata Hospital, Chieti, 66100, Italy.; ^5^San Rossore Breast Unit, Pisa 56122, Italy.; ^6^Institute of Dermatology, F. Policlinico Gemelli IRCSS, Università Cattolica del Sacro Cuore, Rome, Italy.

**Keywords:** Adipose Stem Cells, Mesenchymal Stem Cells, Stem Cell Therapy, COVID-19, SARS-CoV-2, Coronavirus therapy

## Abstract

Mesenchymal Stem Cells (MSCs), and Adipose-Derived Mesenchymal Stem Cells (AD-MSCs) have been used for many years in regenerative medicine for clinical and surgical applications. Additionally, recent studies reported improved respiratory activity after intravenous administration of MSCs into patients affected by coronavirus disease 2019 (COVID-19) caused by the Coronavirus 2 (SARS-CoV-2) suggesting their role as anti-viral therapy. Severe COVID-19 patients usually progress to acute respiratory distress syndrome, sepsis, metabolic acidosis that is difficult to correct, coagulation dysfunction, multiple organ failure, and even death in a short period after onset. Currently, there is still a lack of clinically effective drugs for such patients. The high secretory activity, the immune-modulatory effect, and the homing ability make MSCs and in particular AD-MSCs both a potential tool for the anti-viral drug-delivery in the virus microenvironment and potential cellular therapy. AD-MSCs as the most important exponent of MSCs are expected to reduce the risk of complications and death of patients due to their strong anti-inflammatory and immune-modulatory capabilities, which can improve microenvironment, promote neovascularization and enhance tissue repair capabilities. In this literature review, the role of regenerative strategies through MSCs, AD-MSCs, and adipocyte-secreted exosomal microRNAs (A-SE-miRs) as a potential antiviral therapy was reported, comparing the results found with current research progress on drugs and vaccines in COVID-19 disease.

For more than 10 years, several investigators have shown the regenerative impact of Mesenchymal Stem Cells (MSCs) in damaged organs and tissues. The International Society for Cellular Therapy (ISCT) [1] suggested four parameters to define MSCs:

(1) MSCs are disc-adherent in standard cultures.
(2) MSCs differentiate in adipocytes, chondroblasts, and osteoblasts.
(3) MSCs express CD73, CD90, and CD105.
(4) MSCs don't express CD11b, CD14, CD19, CD34, CD45, CD79, c-kit, and human leukocyte antigen-DR.

The Stromal Vascular Fraction Cells (SVFs) and adipose-derived mesenchymal stem cells (AD-MSCs) both contained in the Stromal Vascular fraction (SVF) portion, meet the majority of the ISCT’s criteria for MSCs [1]. The SVF provides a rich source of AD-MSCs that can be easily isolated from human adipose tissue (HAT). Generally, each mL of HAT offers 100,000-300,000 SVFs of which 1%-3% is constituted by AD-MSCs (1,000-9,000 AD-MSCs/mL) representing a potential source of antiviral drugs in Coronavirus disease (COVID-19) patients as previously suggested by Gentile et al. [2]. The SVFs and related AD-MSCs were used for many years in regenerative medicine without however any focus on their potential anti-viral roles. The anti-inflammatory and immune-modulatory activities of SVFs and AD-MSCs make them consider also, a potential cellular therapy in COVID-19 as already displayed by the use of MSCs in seven COVID-19 patients [3]. These effects were confirmed by the increased peripheral lymphocytes amount, the decline in the C-reactive protein, and waning of over-activated cytokine-secreting immune cells (CXCR3+CD4+ T cells, CXCR3+CD8+ T cells, and CXCR3+ NK cells) into the blood of COVID-19 patients, by mean 4.5 days later the MSCs intravenous infusion [3]. Moreover, 10 x RNA-sequencing analysis displayed that infused MSCs were negative for angiotensin I converting enzyme-2 receptor (ACE2) and transmembrane serine protease 2 (TMPRSS2), which confirmed that these cells were free from COVID-19 infection. The possible implications of MSCs as anti-viral therapy have been cited by also the Kyoto Encyclopedia of Genes and Genomes [3].

As known, at the end of 2019, novel Coronavirus was diagnosed for the first time in Wuhan City, Hubei Province. Since the outbreak of the disease, the number of confirmed cases has increased dramatically in a short period of time [3]. The International Virus Classification Committee named it Severe Acute Respiratory Syndrome Related Virus 2 (SARS-CoV-2), and World Health Organization (WHO) named COVID-19 the Coronavirus disease caused by SARS-CoV-2 [3]. SARS-CoV-2 is similar to severe acute respiratory syndrome-related coronavirus (SARS-CoV) that broke out in 2003 in China, but it has a longer incubation period and stronger infectivity [3].

As of July 9, 2020, the WHO has reported 12,415,672 confirmed cases worldwide, with 557,925 deaths, and 7,241,644 recovered (www.worldometers.info/coronavirus/).

Of these numbers, the WHO has confirmed 4,616,103 currently infected patients where 4,557,416 (99%) are in Mild Condition and 58,687 (1%) in serious or critical conditions (www.worldometers.info/coronavirus/).

For this reason, it is not more possible to accept the idea, that for a viral pandemic, at the current day in July 2020, it is necessary to stay at home to avoid contagion, like Middle Ages, or it is necessary to be hospitalized, in intensive therapy to continue to breathe.

During the last years, MSCs, and AD-MSC-based cellular therapies have been tested in several clinical settings like chronic ischemic cardiomyopathy, inflammatory bowel disease, rheumatoid arthritis, soft-tissue sarcoma reconstruction, graft-versus-host disease, and outcomes of reconstruction after breast cancer [2, 4]. Just a few weeks ago, the use of clinical-grade MSCs in seven COVID-19 patients via intravenous infusion, has been published, reporting exceptional results, with the final healing of patients treated [3].

In this current review, the role of regenerative strategies through MSCs, focusing on AD-MSCs, and adipocyte-secreted exosomal microRNAs (A-SE-miRs) as a potential antiviral therapy was reported, comparing the results found with current research progress on drugs and vaccines in COVID-19 disease.

## MATERIALS AND METHODS

### Search strategy

This systematic review was performed in accordance with the statements and guidelines of the Preferred Reporting Items of Systematic Reviews and Meta-analyses (PRISMA) (www.prisma-statement.org). The search was conducted in accordance with the PRISMA guidelines and the Cochrane handbook. A multistep search of the PubMed, MEDLINE, Embase, PreMEDLINE, CINAHL, PsycINFO, Clinicaltrials.gov, Scopus, and Cochrane databases was performed using different keywords *(“Adipose Stem Cells COVID-19”*, *“Mesenchymal Stem Cells COVID-19”*, *“COVID-19 Stem Cell Therapy”*, *“COVID-19 drugs”*, *“COVID-19 Stem Cells”*). In total 1.023 articles were initially found, COVID-19 drugs (n = 861), Adipose Stem Cells COVID-19 (n = 4), Mesenchymal Stem Cells COVID-19 (n = 30), COVID-19 Stem Cell Therapy (n = 69), COVID-19 Stem Cells (n = 59), as reported in Diagram 1.

### Data Extraction

**Figure 1.**
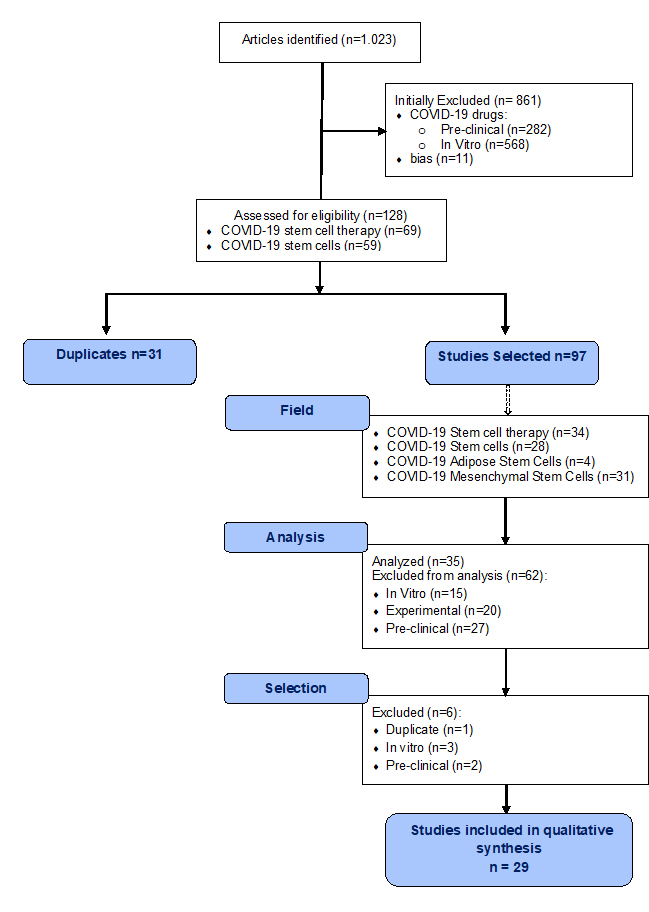
CONSORT (Consolidated Standards of Reporting Trials) flow diagram.

Of these amount, 128 articles focused on stem cells therapy in COVID-19 were initially identified using Prisma Flow. 97 articles published were reviewed after the exclusion of 31 duplicates. Only articles on Adipose Stem Cells (ASCs) and Mesenchymal Stem Cells (MSCs) implications in COVID-19 were considered, and for this reason a total of 62 articles (*in vitro*, experimental, and pre-clinical studies) were excluded. The authors decided to select only article reporting the use of ASCs and MSCs for *in vivo* application in COVID-19 patients. Duplicate studies, *in vitro* and animal studies were discarded screening only relevant articles according to title and abstracts. No time or language limits were adopted. Only 29 articles cited in the text, were analyzed and considered using PRISMA flow (Fig. 1).

Data were independently collected by the first investigator (PG) and checked by the last investigator (SG). Disagreement on collected data was settled by consensus between all the investigators.

The quality of the included studies was independently assessed using two investigators (PG and SG) using the Cochrane Collaboration’s Risk of Bias Assessment tool for RCT while using the Newcastle-Ottawa Scale (www.ohri.ca/programs/clinical_epidemiology/oxford.asp) to evaluate the individual non-randomized studies.

### Aims and outcomes

The primary outcomes were the potential use, safety, and efficacy of ASCs and MSCs use in COVID-19 therapy. Secondary outcomes were side effects. All results collected from the studies were reported with the same measurements retrieved from the papers.

## RESULTS

### Selection of included studies and study characteristics

A total of 1.023 relevant articles were initially identified by searching several online databases. Figure 1 presents the screening and selection process of the eligible articles reporting also the characteristics of the excluded studies. This systematic review included in qualitative synthesis 29 studies. The kind, characteristics, and results of the studies analyzed were presented in Table 1.

**Table 1:** Research progress of COVID-19 therapy based on drugs, vaccines and Mesenchymal Stem Cells.

Drugs	Type	Effects Timing	Biomolecular pathway/indications	Studies	References	Advantages	Disadvantages
Chloroquine and Hydroxy-chloroquine	Antimalaric	< 5 days	Early tretament of severe COVID-19 patients	In Vivo/ Clinical Study	[14, 15]	Early clinical efficacy in severe COVID-19 patients	Not specific drug against SARS-COV-2 Not autologous drug
Remdesivir	Antiviral drug of nucleoside analogs	-	Inhibition of natural nucleoside triphosphate (NTP) to prevent virus RNA synthesis and virus replication	In vitro/ Animal models/ Phase II and Phase III clinical trials ongoing	[7, 16]	Antiviral targeted against SARS-COV-2	Ongoing Clinical trials Not autologous drug Antiviral not specific against SARS-COV-2 Not autologous drug
Nucleic acid Vaccines	Vaccines	-	-	In Vivo/ Clinical study	NCT04283461	Prevention	Not yet final clinical trails results
Inactivated vaccine	Vaccines	-	-	Animal Models	-	Prevention	No yet cllinical trials
Recombinant protein vaccine	Vaccines	-	-	Ready for Clinical trial	-	Prevention	Not yet final clinical trials results
Recombinant virus vector vaccine	Vaccines	-	-	Adenovirus vector vaccine clinical trials Two Lentivirus vector vaccine Clinical trials	NCT04313127 NCT04299724, NCT04276896	Prevention Prevention Prevention	Ongoing clinical trials Ongoing clinical trials Ongoing clinical trials
MSCs	Antiviral	mean 4.5 days	Secreting antibacterial peptides and proteins (AMPs), indoleamine 2,3-dioxygenase (IDO), IL-17/ Activating a large number of anti-virus genes, such as IFITM gene, which can encode protein structures that prevent viruses from invading cells/ regulating the dynamic coordination of pro-inflammatory and anti-inflammatory elements of the patient’s immune system and promoting the activity of phagocytes	In Vivo/ Clinical/ In Vitro/	[19-22]	Early clinical efficacy in severe COVID-19 patients Autologous drug Autologous source of donor tissue Allogeneic use No svere immune reaction	Ongoing clinical trial Not specific drug against SARS-COV-2
AD-MSCs	Antiviral/ Delivery drugs	-	Anti-inflammatory (IL-10), immune-modulatory (TGFß-1), (HGF), (INF-γ), and pro-angiogenic activities (VEGF), (PDGF)/ Transition from inflammatory macrophage phenotype M1 to the anti-inflammatory and wound healing M2 phenotype/ Inhibition of ECM degradation through the increased binding of MMPs and secretion of TIMPs/ Delivery drug	In Vitro/ In Vivo Clinical trial ongoing	[2, 26, 27]	Early clinical efficacy in severe COVID-19 patients Autologous drug Autologous source of donor tissue Allogeneic use Potential delivery drugs No svere immune reaction Potential aereosol administration	Ongoing clinical trial Not specific drug against SARS-COV-2

### Research progress of COVID-19 therapy based on drugs and vaccines

Both Chloroquine and Hydroxychloroquine have been proved to have certain effects on the treatment of patients with COVID-19 (Table 1). A study showed that Hydroxychloroquine has better effects in the early treatment of severe patients (< 5 days) [14, 15]. Some scholars claimed that Remdesivir (RDV), as an antiviral drug of nucleoside analogs, can add virus RNA dependent RNA polymerase (RdRp) through competitive inhibition of natural nucleoside triphosphate (NTP) to prevent virus RNA synthesis and thus inhibit virus replication (Table 1). RDV has shown good anti-MERS-CoV, SARS-CoV, and SARS-CoV-2 activities *in vitro* studies and animal models, indicating that RDV can be used as a potential anti-COVID-19 drug, but its safety and effectiveness still need to be verified by phase II and phase III clinical trials [7, 16].

Pharmaceutical companies and epidemic prevention agencies all over the world have begun to develop the COVID-19 vaccine, but because the vaccine must have sufficient scientific basis and sufficient safety, its research and development cycle may take months to years. Based on the drug research and development experience of MERS-CoV and SARS-CoV, searching for new potential drugs from past drugs has become the current main strategy due to its low research cost and short research and development cycle. At present, the recombinant protein vaccine has just been approved to start clinical trials, the inactivated vaccine is in the stage of establishing animal infection models, the nucleic acid vaccine has carried out clinical trials (NCT04283461) (https://clinicaltrials.gov/ct2/show/NCT04283461), and the recombinant virus vector vaccine has started adenovirus vector vaccine clinical trials (NCT04313127) (https://clinicaltrials.gov/ct2/show/NCT04313127) and two other lentivirus vector vaccine clinical trials (NCT04299724 https://clinicaltrials.gov/ct2/show/NCT04299724), NCT042 76896 https://clinicaltrials.gov/ct2/show/NCT04276896) (Table 1). However, due to the lack of animal models that can effectively evaluate *in vivo* efficacy and the diversity and mutability of coronaviruses, the research and development of vaccines and new drugs are still facing approved [17].

### The antiviral effects of MSCs

Current research has confirmed that MSCs can exert their antiviral effects versus COVID-19 via directs and indirect mechanisms. MSCs can produce a direct anti-viral effect by secreting antibacterial peptides and proteins (AMPs), indoleamine 2,3-dioxygenase (IDO), IL-17 and other molecules, and unlike somatic cells that produce interferon during virus invasion and then activate hundreds of genes that resist virus infection, MSCs can continuously activate a large number of anti-virus genes independent of interferon, such as IFITM gene, which can encode protein structures that prevent viruses from invading cells [19] (Table 1). Additionally, MSCs can also exert an indirect antiviral effect by regulating the dynamic coordination of pro-inflammatory and anti-inflammatory elements of the patient’s immune system and promoting the activity of phagocytes [20] (Table 1). Researchers have also confirmed the immune-regulation and antibacterial and antiviral values of MSCs *in vitro* sepsis model, ARDS model, and alveolar epithelial fibrosis model [21, 22]. So far MSCs have been found to secrete at least four AMPs: antibacterial peptide LL-37 (Cathelicidin LL-37), human defensin-2 (Human defensin-2), hepcidin, and lipocalin-2. These AMPs mediate the cell-killing process by cell killing, inhibiting the synthesis of essential proteins, DNA and RNA of infected cells, and interacting with certain targets in infected cells, and play an active regulatory role in the infection and inflammatory progress of patients with COVID-19 [20].

### AD-MSCs and their A-SE-miRs as cellular weapons in the inflammatory process

The AD-MSCs offer several advantages compared to other MSCs. First of all, they can be harvest through easy, quick, and not invasive liposuction in the abdomen region, flanks, and/or thighs using three millimeters (mm) diameter cannula. They have cell yield more than one thousand-fold higher when compared to Bone-Marrow MSCs (BM-MSCs) [23, 24] and Umbilical-Cord MSCs (UC-MSCs) [23], presenting a longer life-span, higher proliferative ability, shorter doubling time.

All these features make AD-MSCs both optimal vehicles to delivery-drugs and real cellular weapons.

The exosomes are membrane-bound extracellular vesicles (EVs) 30 to 150 nanometers (nm) in size, produced in the endosomal compartment of most eukaryotic cells, and they are released by cells working as intercellular transmitters of mRNA, microRNAs (miRs), and proteins [24]. They are found in several tissues and also in blood, urine, and cerebrospinal fluid but a particular and growing interest has been displayed for those EVs present in fats [24], that here calling AD-MSC-secreted exosomes (A-SE).

As known, in COVID-19 patients, the pneumonia is induced by a cytokines storm produced by SARS -CoV-2 thanks to using a specific cell entry receptor-ACE2 [3]. The cytokines storm represents an important inflammatory activity produced as a human immune response to the SARS-CoV-2 attack.

As described, SVFs and AD-MSCs present anti-inflammatory, immune-modulatory, and pro-angiogenic activities. In fact, they secrete pro-angiogenic factors, such as Vascular Endothelial Growth Factor (VEGF), platelet-derived growth factors (PDGF), inducing proliferation of endothelial cells, promoting the vascularization, providing physical extracellular matrix (ECM) guidance cues that promote endothelial sprouting [2] (Table 1). Their immune-modulating proprieties are driven by transforming growth factor beta-1 (TGFß-1), hepatocyte growth factors (HGF), and interferon-γ (INF-γ) [2]. Their anti-inflammatory activity is mediated by IL-10. Additionally, AD-MSCs may induce the transition from inflammatory macrophage phenotype M1 to the anti-inflammatory and wound healing M2 phenotype [25]. Finally, AD-MSCs have been demonstrated to inhibit ECM degradation through the increased binding of matrix metallopeptidases (MMPs) and secretion of tissue inhibitors of MMPs (TIMPs) [26] (Table 1).

All these activities (anti-inflammatory/immune-modulatory/immune-suppressive) and also the early establishment of new micro-capillary networks, which deliver the proper nutrients and oxygen, might explain the exceptional results observed during MSCs infusion in COVID-19 patients.

The high secretory activity makes also SVFs and AD-MSCs, a potentially suitable vehicle for the delivery of drugs molecules in the cellular microenvironment, with the potential aim to regenerate damaged tissue via their exosomes, and related miRs [27]. These results provide important insights into A-Se-MiRs function and could suggest their use as anti-viral. On the basis of the mentioned above concepts, ASCs treatments may reduce the demand for critical hospital resources [29].

**Figure 2.**
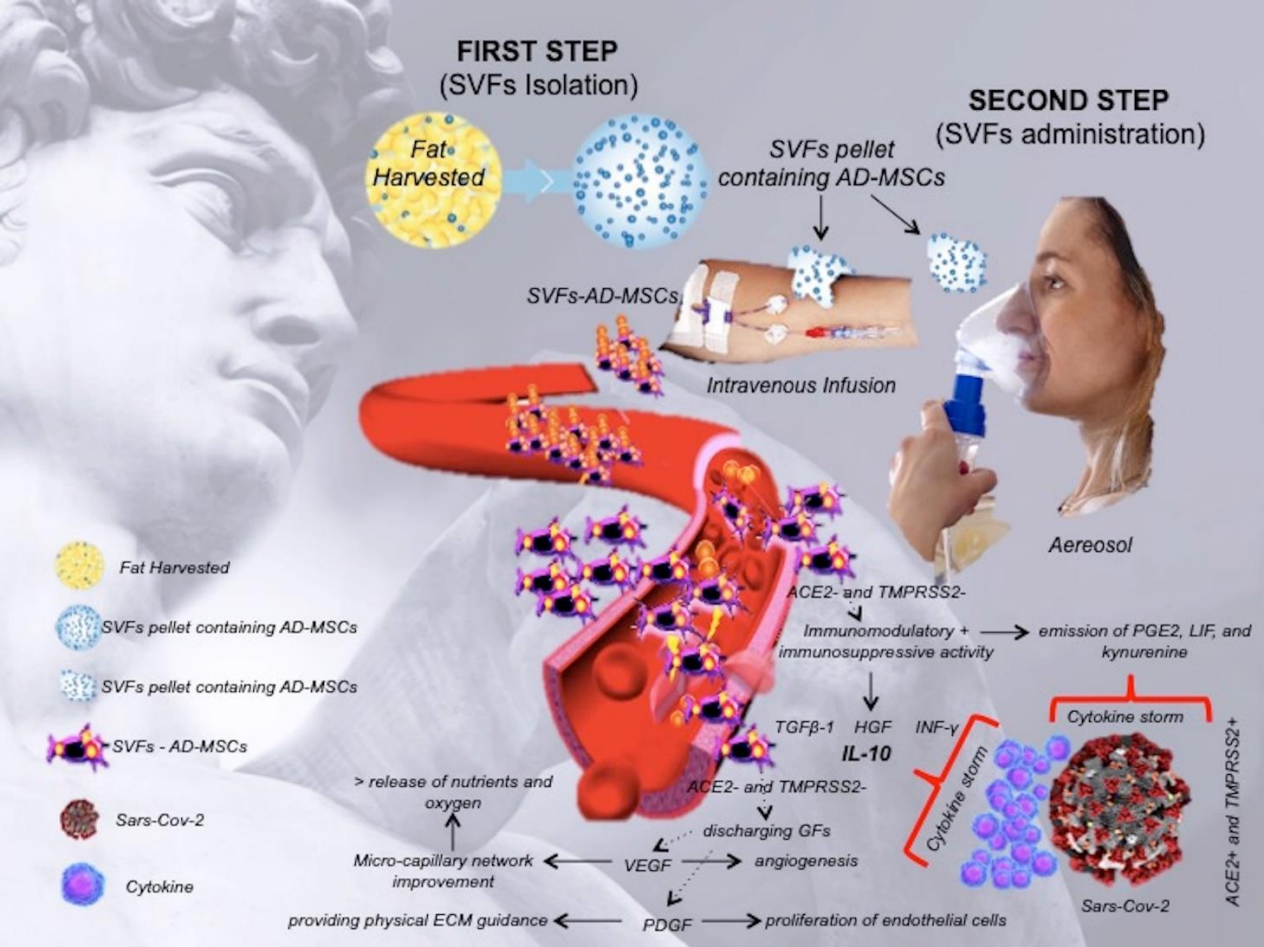
Graphical illustration of the anti-viral activity of SVFs and AD-MSCs via intravenous infusion and aerosol administration.

A graphical illustration of the anti-viral activity of SVFs and AD-MSCs is synthetically reported in Figure 2.

### Strategies of the AD-MSCs and MSCs use in COVID-19

Zhao’s group [3] described an intravenous infusion of 1 × 10^6^MSCs per Kg of weight, in patients affected by COVID-19, suggesting a total amount of 75 × 10^6^ MSCs for each patient, hypothesizing a weight mean of 75Kg (data not published). Applying procedures based on minimal manipulation of HAT could be necessary to collect a 500-1000mL average of fat, to generate 50 × 10^6^ - 100 × 10^6^AD-MSCs.

Using enzymatic digestion of HAT, it is possible to obtain approximately 250,000 ± 34,782 nucleated SVFs cells per each mL of HAT [2]. In this case, 25 × 10^6^ nucleated SVFs cells can be obtained by 100mL of HAT, and 300mL of HAT would be the right amount to obtain an average of 75 × 10^6^ cells.

Alternatively, using only a few each mL of HAT for each patient (5-10mL) would be necessary to perform extensive manipulation via Good Manufacturing Practices (GMP) lab, to obtain the same quantity of cells in form of the cellular product like as which used by Zhao’s group [3].

All the above-mentioned procedures of HAT manipulation, aimed to obtain an SVFs pellet containing AD-MSCs, are regulated by the European rules (1394/2007 EC) and European Medical Agency (EMA) / Committee for Advanced Therapies (CAT) recommendations (20 June 2014, EMA/CAT/600280/ 2010 Rev 1) [2, 27].

### Suggested therapies in COVID-19 patients, using AD-MSCs and their related A-SE-miRs

It is possible to divide two different eventual applicative protocols: a) emergency protocol; b) consolidated administration

In the first case, indicated for the COVID-19 treatment, as described previously, could be possible immediately, buy or have free, the MSCs as AD-MSCs by:

– Food and Drug Administration (FDA) approved labs and/or tissues bank;
–GMP laboratory;
–EMA approved labs or tissues bank.

During this first emergency step, it could be possible to start the AD-MSCs infusion, in patients at the same time at the conventional therapy.

In the second case, it could be possible to start with the MSCs production (AD-MSCs prevalently), using an autologous or allogeneic cellular product. In the last case, it could be possible to donate HAT to GMP, EMA, or FDA Laboratory or bank to isolate AD-MSCs and re-infuse the cellular product obtained, in COVID-19 patients.

It is necessary to specify that these procedures are possible if performed and authorized by the GMP lab or EMA in Europe and by the FDA in the United States.

The rationale of the present work is to suggest the possibility to use autologous or allogeneic AD-MSCs intravenously or directly through a ventilation mask (aerosol).

### Safety of MSCs Treatment

The most important thing is the quality of MSCs and AD-MSCs, which should come from GMP laboratories that meet the standards of EMA, and FDA. Since most of the injected MSCs and AD-MSCs should come from allogeneic tissues, we should always be alert for allergic reactions in patients. There have been reports of serious complications caused by the improper application of MSCs in the past [28]. Donor-specific antibodies have been observed in 19-34% of patients receiving allogeneic AD-MSCs infusion suggesting that a cellular response can occur [29]. On the other hand, no clinical immune reactions have been seen in studies that have used intravenous AD-MSCs (1×10^6^ ASCs/kg) as reported in a recent review [29].

First-line doctors should formulate individualized treatment plans according to the patient’s situation, such as cell dosage, cell suspension concentration, and infusion speed, so as to ensure the maximum curative effect.

### Current Clinical trials registered on AD-MSCs use in COVID-19 patients

Currently, at July 9, 2020, are registered and ongoing five clinical trials https://clinicaltrials.gov/ct2/results?cond=COVID-19&term=Adipose+stem+cells&cntry=&state=&city=&dist= based on the administration of AD-MSCs in COVID-19 patients and of these three are authorized, as followed reported:

-Clinical study for the prophylactic efficacy of AD-MSCs against COVID-19 (Trial ID NCT04428801 https://clinicaltrials.gov/ct2/show/record/NCT04428801?term=Adipose+stem+cells&cond=COVID-19&draw=2&rank=1);

- A Randomized, placebo-controlled, double-blind, single center, efficacy and safety study of Allogeneic Hope Biosciences adipose derived mesenchymal stem cells (HB-adMSCs) for the Treatment of COVID-19 (Trial ID NCT04362189) (https://clinicaltrials.gov/ct2/show/NCT04362189?term=Adipose+stem+cells&cond=COVID-19&draw=2&rank=6);

- Study with stem cells from allogenic adipose tissue, in patients with coronavirus severe pneumonia (Trial ID EUCTR2020-001364-29-ES) (www.clinicaltrialsregister.eu/ctr-search/search?query= eudract_number:2020-001364-29);

- Clinical trial of administration of MSC to patients with respiratory distress type COVID-19 (Trial ID EUCTR2020-001266-11-ES) (www.clinicaltrialsregister.eu/ctr-search/search?query=eudract_number:2020-001266-11);

- Clinical study to assess the safety and preliminary efficacy of HCR040, a drug based on mesenchymal stem cells, in patients with acute respiratory distress syndrome. (included patients COVID-19) (Trial ID EUCTR2019-002688-89-ES) (www.clinicaltrialsregister.eu/ctr-search/search?query=eudract_number:2019-002688-89).

Two clinical trials (EUCTR2020-001364-29-ES and EUCTR2020-001266-11-ES) were registered in April 2020, after the pandemic situation produced by COVID-19 but the last one (EUCTR2019-002688-89-ES) based on the possibility *“To assess the feasibility, safety, and tolerability of the administration of HCR040, a drug whose active substance is HC016, allogeneic adipose-derived adult mesenchymal stem cells expanded and pulsed with H2O2, in patients with acute respiratory distress syndrome. Included COVID-19 patients”* was registered on July 26, 2019, and started at 2019-12-10 (www.clinicaltrialsregister.eu/ctr-search/search?query=eudract_number:2019-002688-89), before the pandemic. How is it possible? No questions are available, but it is an interesting coincidence.

## DISCUSSION

### COVID-19 disease

COVID-19 is an acute self-limiting disease. The clinical manifestations of patients are complex and varied from no obvious symptoms to severe respiratory failure that mechanical ventilation support is required. Severe patients are also prone to acute respiratory distress syndrome (ARDS), coagulation dysfunction, metabolic acidosis difficult to correct, septic shock, and multiple organ failure (MOD) [5]. Huang et al. [6] were the first to report the clinical situation of patients in China. Arentz et al. [7] analyzed 21 critically ill patients with COVID-19 in Washington State, USA. The initial symptoms were chest tightness and shortness of breath (76%), high fever (52%), cough (48%), and most patients (86%, n=18) had basic chronic diseases when they were admitted to hospital, mostly kidney diseases and heart failure. 81% (n=17) patients need to be transferred to ICU within 24 hours after admission. All 15 patients with ARDS need to receive mechanical ventilation assisted respiratory therapy, of which 8 (53%) patients progress to severe ARDS within 72 hours. The medical staff around the world soon realized that there was currently no specific treatment for COVID-19. The treatment strategy for severe patients was to prevent and treat complications on the basis of symptomatic treatment while actively treating basic diseases and preventing secondary infection.

Blood purification, artificial membrane lung, and serum perfusion of convalescent patients lack sufficient effectiveness for disease treatment, and targeted vaccines cannot be developed in the short term. As a sudden major global public health event, researchers are urgently needed to develop safe and effective treatment methods. At present, cell-based therapy has been formally incorporated into the diagnosis and treatment guidelines or consensus for diseases including lung, cardiovascular, liver, and kidney [8, 9], and there are also multi-center successful clinical cases of umbilical cord stem cell therapy for critically ill patients in China [3]. On February 15, 2020, Professor Zhang Xinmin, director of the China Biological Center of the Ministry of Science and Technology held a press conference at the joint defense and control mechanism of the State Council. He reported that MSCs technology can improve microcirculation, promote endogenous repair and relieve ARDS symptoms by inhibiting the over-activation of the immune system of patients with COVID-19, and affirmed its effect in the treatment of severe patients [10].

### The pathogenesis of SARS-CoV-2 and inflammatory process

Some studies have shown that SARS-CoV-2 is pathogenic through the specific recognition of its Spike protein (S protein) and ACE-2. Cells with positive ACE-2 expression are more susceptible (such as SARS-2003 virus, but its affinity with ACE-2 is only 5% - 10% of SARS-CoV-2) [11]. Another study showed that the intracellular TMPRSS2 has the ability of specific recognition and binding with the S protein of SARS-CoV-2, which plays an important role in the process of virus infection and transmission, ending in the inflammatory cytokine storm [12]. Commonly, the inflammatory stage is triggered by a pathogen resulting in the molecules patterns (pathogen-associated molecular patterns -PAMPs-) release, that ligates and activate NOD-like receptors (NLRs), toll-like receptors (TLRs), C-type lectin receptors (CLRs) or other receptors on endothelial cells, mast cells, macrophages and interstitial fibroblasts [18]. The receptors activation induces the discharge of cytokines, chemokines, and growth factors (GFs) triggering neutrophils and monocytes recruitment [18].

The monocytes/macrophages (MM) during the passage from inflammatory (M1) to healing stage (M2) produce a big number of cytokines and GFs that promoting the multiple cell types proliferation involved in tissue repair [18]. As preliminary reported by Leng et al [3], not seems to be clear the reason for continuing cytokine storm in COVID-19 patients, without the transition between the inflammatory to the healing stage. For this reason, it appears necessary to act inducing this transition since inflammatory to the healing stage via MSCs, AD-MSCs, and their A-SE-miRs.

ACE-2 is widely distributed on the cell surface of human except for bone marrow, lymph nodes, thymus, spleen, and immune cells, while TMPRSS2 is highly expressed in alveolar type II epithelial cells and capillary endothelial cells [13]. Therefore, COVID-19 pathogens entering the patient’s blood circulation can be widely spread in a short period of time, and lead to organ damage other than lungs such as acute kidney injury, myocardial injury, shock, and MOD. In the analyzed studies, a history of organ dysfunction was identified as the predictor of severe disease. In addition, the pathological changes in the multiorgan tissues of COVID-19 patients resemble those observed in SARS and MERS infection. Coronaviruses not only induce direct organ damage but also aggravate the injury through proinflammatory methods, as previously discussed.

On the basis of the data evaluated, it is possible to affirm that SARS-CoV-2 shared some similarities with SARS-CoV and/or MERS-CoV including genomic sequence, origin, entry receptor, clinical features, risk factors, and pathological processes. For this reason, the clinical experience from SARS and MERS can provide some guidance regarding therapy strategies in COVID-19 patients while the world waiting for the definitive vaccine and the specific treatment.

This article reviewed, at the current day, the research progress on stem cell therapy in COVID-19 patients, and in particular focused on the clinical research status of MSCs and AD-MSCs biotechnology, reporting also an update on drugs and vaccines, so as to provide a reference for frontline clinical and scientific researchers.

### Limitations

Our systematic review has some limitations. First, most of the included studies are based on a little group of patients treated showing a low level of evidence. Second, during the period of publication of this systematic review (July 2020), most therapies are yet ongoing, and vaccines are testing, remained which results in missing data.

### Conclusion

It is imperative that research on MSCs and AD-MSCs be conducted in a transparent manner, according to established precedents for clinical research of new therapies in critical illness, pushing the rapid and full data sharing, once they are adequately quality controlled for release.

In this sense, MSCs, AD-MSCs, and theirs A-SE-miRs could be considered a potential and immediate anti-viral therapy for COVID-19 patients, on the basis of their anti-inflammatory, and immune-suppressive activity, contrasting directly the cytokine storms causing pneumonia and other organs disease. In particular, these cellular-therapies could be considered, in severe COVID-19 patients that do not respond to the administered drugs. Vaccines are not yet ready, and some drugs indicated for other purposes are testing in COVID-19 patients. For this reason, it could be necessary to start definitively with cellular therapy based on intravenous infusion and/or aerosol of MSCs and AD-MSCs.
